# Supplementary material for: Postsynaptic adaptations in direct pathway muscarinic M4-receptor signaling follow the temporal and regional pattern of dopaminergic degeneration
Source: NPJ Parkinsons Dis. 2025 Jul 1;11:186. doi: 10.1038/s41531-025-01047-3 (PMC12219286; doi:10.1038/s41531-025-01047-3)
Supplement: Supplementary file 1 — Supplementary information [file 41531_2025_1047_MOESM1_ESM.pdf]

Supplemental Table S1 - Extended statistical data

Figure	Panel	Variables and units	Conditions	Mean	SEM	n	N	Statistical test	Statistic-value	p-value	(*)	Post-hoc test	Post-hoc comparisons	p-value	(*)
		DSt	Saline	1.00	0.00		4						Saline vs. 6-OHDA LD	p<0.0001	****
		Fluorescence ratio	6-OHDA LD	0.44	0.04		6	Ordinary One-Way ANOVA	F(2, 12)=189.2	p<0.0001	****	Tukey	Saline vs. 6-OHDA HD	p<0.0001	****
	C	(vs contralateral hemisphere)	6-OHDA HD	0.09	0.00		5						6-OHDA LD vs. 6-OHDA HD	p<0.0001	****
		SNc	Saline	1.08	0.04		4						Saline vs. 6-OHDA LD	p<0.0001	****
		Fluorescence ratio	6-OHDA LD	0.43	0.05		6	Ordinary One-Way ANOVA	F(2, 12)=141.0	p<0.0001	****	Tukey	Saline vs. 6-OHDA HD	p<0.0001	****
1		(vs contralateral hemisphere)	6-OHDA HD	0.10	0.01		5						6-OHDA LD vs. 6-OHDA HD	p=0.0001	***
		Saline - Fluorescence ratio	DLS	1.04	0.04		4	Paired t-test	t(3)=0.4222	p=0.7013	n.s.				
		(vs contralateral hemisphere)	DMS	1.02	0.02		4								
	D	6-OHDA LD - Fluorescence ratio	DLS	0.34	0.03		6	Paired t-test	t(5)=3.546	p=0.0165	*				
		(vs contralateral hemisphere)	DMS	0.52	0.06		6								
		6-OHDA HD - Fluorescence ratio	DLS	0.09	0.00		5	Paired t-test	t(4)=1.884	p=0.1327	n.s.				
		(vs contralateral hemisphere)	DMS	0.09	0.01		5								
		Paw use (% contralateral) vs													
		DSt TH+-fiber loss (%)					24	Pearson correlation	r=-0.8376	p<0.0001	****				
		DLS TH+-fiber loss (%)					24	Pearson correlation	r=-0.8471	p<0.0001	****				
		DMS TH+-fiber loss (%)					24	Pearson correlation	r=-0.8308	p<0.0001	****				
	A	Latency to fall (s) vs													
		DSt TH+-fiber loss (%)					24	Spearman correlation	r=-0.8232	p<0.0001	****				
2		DLS TH+-fiber loss (%)					24	Spearman correlation	r=-0.7694	p<0.0001	****				
		DMS TH+-fiber loss (%)					24	Spearman correlation	r=-0.8048	p<0.0001	****				
			Saline	51.67	1.36		9						Saline vs. 6-OHDA LD	p=0.0072	**
	B	Paw use (% contralateral)	6-OHDA LD	33.77	2.57		13	Kruskal-Wallis	H=23.66	p<0.0001	****	Dunn	Saline vs. 6-OHDA HD	p<0.0001	****
			6-OHDA HD	18.29	4.07		14						6-OHDA LD vs. 6-OHDA HD	p=0.1443	n.s.
			Saline	288.70	4.10		9						Saline vs. 6-OHDA LD	p=0.0114	*
	C	Latency to fall (s)	6-OHDA LD	210.80	20.54		13	Ordinary One-Way ANOVA	F(2, 36)=20.99	p<0.0001	****	Tukey	Saline vs. 6-OHDA HD	p<0.0001	****
			6-OHDA HD	134.40	14.47		17						6-OHDA LD vs. 6-OHDA HD	p=0.0032	**
			Saline	611.30	41.66	21	6						Saline vs. 6-OHDA LD	p<0.0001	****
		M4-IPSC amplitude (pA) DLS	6-OHDA LD	398.50	33.88	29	11	Ordinary One-Way ANOVA	F(2, 68)=22.58	p<0.0001	****	Tukey	Saline vs. 6-OHDA HD	p<0.0001	****
	C		6-OHDA HD	271.30	22.59	21	7						6-OHDA LD vs. 6-OHDA HD	p=0.0251	*
			Saline	411.40	38.06	21	11						Saline vs. 6-OHDA LD	p>0.9999	n.s.
		M4-IPSC amplitude (pA) DMS	6-OHDA LD	395.20	41.45	29	9	Kruskal-Wallis	H=14.40	p=0.0007	***	Dunn	Saline vs. 6-OHDA HD	p=0.0027	**
			6-OHDA HD	249.50	24.25	36	11						6-OHDA LD vs. 6-OHDA HD	p=0.0074	**
			eStim intensity x Treatment					Two-Way RM ANOVA with	F(10, 340)=6.003	p<0.0001	****				
		M4-IPSC amplitude (pA) DLS	eStim intensity					Geisser Greenhouse's	F(1.842, 125.3)=201.8	p<0.0001	****	Holm-Šídák			
		vs eStim intensity (µA)	Treatment					correction	F(2, 68)=25.80	p<0.0001	****				
			Subject						F(68, 340)=22.31	p<0.0001	****				
			Saline	638.18	40.34	21	6						Saline vs. 6-OHDA LD	p=0.0005	***
		30 µA	6-OHDA LD	417.87	37.83	29	11						Saline vs. 6-OHDA HD	p<0.0001	****
			6-OHDA HD	303.11	26.26	21	7						6-OHDA LD vs. 6-OHDA HD	p=0.0163	*
			Saline	611.32	41.66	21	6						Saline vs. 6-OHDA LD	p=0.0006	***
		25 µA	6-OHDA LD	398.53	33.88	29	11						Saline vs. 6-OHDA HD	p<0.0001	****
			6-OHDA HD	271.26	22.59	21	7						6-OHDA LD vs. 6-OHDA HD	p=0.0031	**
			Saline	563.17	40.91	21	6						Saline vs. 6-OHDA LD	p=0.0011	**
		20 µA	6-OHDA LD	371.75	30.06	29	11						Saline vs. 6-OHDA HD	p<0.0001	****
			6-OHDA HD	237.98	25.18	21	7						6-OHDA LD vs. 6-OHDA HD	p=0.0013	**
			Saline	503.93	36.12	21	6						Saline vs. 6-OHDA LD	p=0.0006	***
		15 µA	6-OHDA LD	321.18	28.93	29	11						Saline vs. 6-OHDA HD	p<0.0001	****
			6-OHDA HD	205.64	22.89	21	7						6-OHDA LD vs. 6-OHDA HD	p=0.003	**
			Saline	421.22	38.21	21	6						Saline vs. 6-OHDA LD	p<0.0001	****
		10 µA	6-OHDA LD	207.62	23.90	29	11						Saline vs. 6-OHDA HD	p<0.0001	****
			6-OHDA HD	150.33	22.03	21	7						6-OHDA LD vs. 6-OHDA HD	p=0.0844	n.s.
			Saline	257.13	30.25	21	6						Saline vs. 6-OHDA LD	p<0.0001	****
3		5 µA	6-OHDA LD	61.80	14.63	29	11						Saline vs. 6-OHDA HD	p<0.0001	****
	D		6-OHDA HD	85.62	16.12	21	7						6-OHDA LD vs. 6-OHDA HD	p=0.2797	n.s.
			eStim intensity x Treatment					Two-Way RM ANOVA with	F(10, 395)=10.13	p<0.0001	****				
		M4-IPSC amplitude (pA) DMS	eStim intensity					Geisser Greenhouse's	F(1.775, 140.2)=251.5	p<0.0001	****	Holm-Šídák			
		vs eStim intensity (µA)	Treatment					correction	F(2, 79)=5.330	p=0.0067	**				
			Subject						F(79, 395)=28.47	p<0.0001	****				
			Saline	476.41	44.62	17	9						Saline vs. 6-OHDA LD	p=0.3762	n.s.
		30 µA	6-OHDA LD	421.63	41.89	29	9						Saline vs. 6-OHDA HD	p=0.0011	**
			6-OHDA HD	267.09	25.27	36	11						6-OHDA LD vs. 6-OHDA HD	p=0.0055	**
			Saline	443.57	43.02	17	9						Saline vs. 6-OHDA LD	p=0.4231	n.s.
		25 µA	6-OHDA LD	395.22	41.45	29	9						Saline vs. 6-OHDA HD	p=0.0016	**
			6-OHDA HD	249.48	24.25	36	11						6-OHDA LD vs. 6-OHDA HD	p=0.0079	**
			Saline	391.54	41.37	17	9						Saline vs. 6-OHDA LD	p=0.4047	n.s.
		20 µA	6-OHDA LD	344.06	38.26	29	9						Saline vs. 6-OHDA HD	p=0.0052	**
			6-OHDA HD	226.97	22.61	36	11						6-OHDA LD vs. 6-OHDA HD	p=0.0227	*
			Saline	316.63	41.34	17	9						Saline vs. 6-OHDA LD	p=0.6167	n.s.
		15 µA	6-OHDA LD	288.69	36.83	29	9						Saline vs. 6-OHDA HD	p=0.0482	*
			6-OHDA HD	195.81	22.41	36	11						6-OHDA LD vs. 6-OHDA HD	p=0.0713	n.s.
			Saline	209.97	46.56	17	9						Saline vs. 6-OHDA LD	p=0.9619	n.s.
		10 µA	6-OHDA LD	207.18	34.40	29	9						Saline vs. 6-OHDA HD	p=0.5103	n.s.
			6-OHDA HD	156.02	20.47	36	11						6-OHDA LD vs. 6-OHDA HD	p=0.5023	n.s.
			Saline	47.39	14.93	17	9						Saline vs. 6-OHDA LD	p=0.8475	n.s.
		5 µA	6-OHDA LD	60.18	15.33	29	9						Saline vs. 6-OHDA HD	p=0.9967	n.s.
			6-OHDA HD	47.32	8.37	36	11						6-OHDA LD vs. 6-OHDA HD	p=0.8475	n.s.
		% change (respect to saline)	DLS	-34.81	5.54	29	11	Mann-Whitney test	U=269	p=0.0181	*				
	E	6-OHDA LD	DMS	-3.93	10.08	29	9								
		% change (respect to saline)	DLS	-55.63	3.70	21	7	Unpaired t-test	t(55)=1.975	p=0.0533	n.s.				
		6-OHDA HD	DMS	-39.36	5.90	36	11								
			Saline	529.90	41.70	12	6						Saline vs. 6-OHDA LD	p=0.0204	*
		Max Oxo-current (pA) DLS	6-OHDA LD	311.50	36.92	11	10	Kruskal-Wallis	H=12.82	p=0.0016	**	Dunn	Saline vs. 6-OHDA HD	p=0.002	**
	B		6-OHDA HD	301.70	43.32	15	7						6-OHDA LD vs. 6-OHDA HD	p>0.9999	n.s.
			Saline	521.80	44.79	14	10						Saline vs. 6-OHDA LD	p=0.8899	n.s.
		Max Oxo-current (pA) DMS	6-OHDA LD	488.20	65.72	8	6	Ordinary One-Way ANOVA	F(2, 33)=7.561	p=0.002	**	Tukey	Saline vs. 6-OHDA HD	p=0.0023	**
			6-OHDA HD	292.10	39.85	14	11						6-OHDA LD vs. 6-OHDA HD	p=0.0295	*
4		% change (respect to saline)	DLS	-41.22	6.97	11	10	Unpaired t-test	t(17)=2.587	p=0.0192	*				
	C	6-OHDA LD	DMS	-6.45	12.59	8	6								
		% change (respect to saline)	DLS	-43.06	8.17	15	7	Mann-Whitney test	U=103	p=0.9486	n.s.				
		6-OHDA HD	DMS	-44.03	7.64	14	11								
		6-OHDA LD - Fluorescence ratio	DLS	0.94	0.02		6	One Sample t-test (vs 1)	t(5)=3.971	p=0.0106	*				
	D	(vs contralateral hemisphere)	DMS	0.92	0.04		6	One Sample t-test (vs 1)	t(5)=2.203	p=0.0788	n.s.				
								Paired t-test (DLS vs DMS)	t(5)=0.3815	p=0.7185	n.s.				
	B	Max ΔF/F (mean frame)	DLS	3.10	0.14	10	5	Unpaired t-test	t(15)=0.5407	p=0.5967	n.s.				
			DMS	3.21	0.14	7	4								
			Saline	0.71	0.06	32	9								
		DLS ΔF/F (mean frame)	6-OHDA LD	0.81	0.05	27	6	Kruskal-Wallis	H=2.838	p=0.2419	n.s.				
	D		6-OHDA HD	0.81	0.03	38	9								
			Saline	0.50	0.04	38	9						Saline vs. 6-OHDA LD	p=0.1618	n.s.
5		DMS ΔF/F (mean frame)	6-OHDA LD	0.61	0.05	28	6	Kruskal-Wallis	H=12.49	p=0.0019	**	Dunn	Saline vs. 6-OHDA HD	p=0.0013	**
			6-OHDA HD	0.68	0.04	42	9						6-OHDA LD vs. 6-OHDA HD	p=0.6193	n.s.
			Saline	1.02	0.07	28	6								
		DLS ΔF/F peak (point scanning)	6-OHDA LD	1.04	0.07	15	4	Kruskal-Wallis	H=3.318	p=0.1903	n.s.				
	E		6-OHDA HD	0.91	0.05	34	5								
			Saline	0.88	0.06	31	7								
		DMS ΔF/F peak (point scanning)	6-OHDA LD	0.93	0.07	15	4	Kruskal-Wallis	H=1.523	p=0.467	n.s.				
			6-OHDA HD	0.99	0.05	27	5								

Figure	Panel	Variables and units	Conditions	Mean	SEM	n	N	Statistical test	Statistic-value	p-value	(*)	Post-hoc test	Post-hoc comparisons	p-value	(*)
5	F	DLS PPR	Saline	0.26	0.02	26	6	Kruskal-Wallis	H=3.066	p=0.2159	n.s.				
			6-OHDA LD	0.23	0.03	15	4								
			6-OHDA HD	0.22	0.02	30	5								
		DMS PPR	Saline	0.29	0.02	31	7	Kruskal-Wallis	H=1.051	p=0.5913	n.s.				
			6-OHDA LD	0.28	0.03	15	4								
			6-OHDA HD	0.26	0.01	28	5								
6	B	Net charge (pA.s) DLS Saline	Baseline	89.59	8.03	16	4	Wilcoxon test	W=136.0	p<0.0001	****				
			Ambenonium	505.30	66.26	16	4								
		Net charge (pA.s) DLS 6-OHDA LD	Baseline	101.50	11.10	20	8	Wilcoxon test	W=210.0	p<0.0001	****				
			Ambenonium	580.50	118.60	20	8								
		Net charge (pA.s) DLS 6-OHDA HD	Baseline	123.40	12.69	15	5	Wilcoxon test	W=120.0	p<0.0001	****				
			Ambenonium	785.70	129.30	15	5								
		Ratio Net Charge DLS	Saline	6.08	0.73	16	4	Kruskal-Wallis	H=1.495	p=0.4736	n.s.				
			6-OHDA LD	5.72	0.94	20	8								
			6-OHDA HD	6.20	0.57	15	5								
		Net charge (pA.s) DMS Saline	Baseline	100.20	18.99	10	6	Wilcoxon test	W=55	p=0.002	**				
			Ambenonium	425.70	109.20	10	6								
		Net charge (pA.s) DMS 6-OHDA LD	Baseline	105.80	11.95	8	6	Paired t-test	t(7)=3.646	p=0.0082	**				
			Ambenonium	411.90	94.03	8	6								
		Net charge (pA.s) DMS 6-OHDA HD	Baseline	60.33	10.64	13	8	Paired t-test	t(12)=5.110	p=0.0003	***				
			Ambenonium	289.30	49.41	13	8								
		Ratio Net Charge DMS	Saline	4.32	0.64	10	6	Ordinary One-Way ANOVA	F(2, 28)=1.898	p=0.1687	n.s.				
			6-OHDA LD	3.57	0.54	8	6								
			6-OHDA HD	5.61	0.85	13	8								
7	B	M4-IPSC amplitude (pA) DLS	Saline	611.30	41.66	21	6	Ordinary One-Way ANOVA	F(2, 65)=12.74	p<0.0001	****	Tukey	Saline vs. 6OHDA LD	p=0.0002	***
			6-OHDA LD	398.50	33.88	29	11						Saline vs. 6OHDA LD + L-DOPA	p<0.0001	****
			6-OHDA LD + L-DOPA	363.80	29.43	18	4						6OHDA LD vs. 6OHDA LD + L-DOPA	p=0.7808	n.s.
		M4-IPSC amplitude (pA) DLS vs eStim intensity (µA)	eStim intensity x Treatment					Two-Way RM ANOVA with Geisser Greenhouse's correction	F(10, 325)=0.8160	p=0.6134	n.s.	Holm-Šídák			
			eStim intensity						F(1.925, 125.1)=240.3	p<0.0001	****				
			Treatment						F(2, 65)=17.47	p<0.0001	****				
			Subject						F(65, 325)=23.57	p<0.0001	****				
		30 µA	Saline	638.18	40.34	21	6						Saline vs. 6OHDA LD	p=0.0005	***
			6-OHDA LD	417.87	37.83	29	11						Saline vs. 6OHDA LD + L-DOPA	p<0.0001	****
			6-OHDA LD + L-DOPA	382.82	30.32	18	4						6OHDA LD vs. 6OHDA LD + L-DOPA	p=0.4735	n.s.
		25 µA	Saline	611.32	41.66	21	6						Saline vs. 6OHDA LD	p=0.0006	***
			6-OHDA LD	398.53	33.88	29	11						Saline vs. 6OHDA LD + L-DOPA	p<0.0001	****
			6-OHDA LD + L-DOPA	363.84	29.43	18	4						6OHDA LD vs. 6OHDA LD + L-DOPA	p=0.4436	n.s.
		20 µA	Saline	563.17	40.91	21	6						Saline vs. 6OHDA LD	p=0.0011	**
			6-OHDA LD	371.75	30.06	29	11						Saline vs. 6OHDA LD + L-DOPA	p<0.0001	****
			6-OHDA LD + L-DOPA	316.25	27.41	18	4						6OHDA LD vs. 6OHDA LD + L-DOPA	p=0.1795	n.s.
		15 µA	Saline	503.93	36.12	21	6						Saline vs. 6OHDA LD	p=0.0006	***
			6-OHDA LD	321.18	28.93	29	11						Saline vs. 6OHDA LD + L-DOPA	p<0.0001	****
			6-OHDA LD + L-DOPA	284.24	27.11	18	4						6OHDA LD vs. 6OHDA LD + L-DOPA	p=0.3566	n.s.
		10 µA	Saline	421.22	38.21	21	6						Saline vs. 6OHDA LD	p=0.0001	***
			6-OHDA LD	207.62	23.90	29	11						Saline vs. 6OHDA LD + L-DOPA	p=0.0002	***
			6-OHDA LD + L-DOPA	213.29	28.15	18	4						6OHDA LD vs. 6OHDA LD + L-DOPA	p=0.8787	n.s.
		5 µA	Saline	257.13	30.25	21	6						Saline vs. 6OHDA LD	p<0.0001	****
			6-OHDA LD	61.80	14.63	29	11						Saline vs. 6OHDA LD + L-DOPA	p<0.0001	****
			6-OHDA LD + L-DOPA	48.03	9.89	18	4						6OHDA LD vs. 6OHDA LD + L-DOPA	p=0.44	n.s.
		M4-IPSC amplitude (pA) DMS	Saline	411.40	38.06	21	11	Kruskal-Wallis	H=4.246	p=0.1197	n.s.				
			6-OHDA LD	395.20	41.45	29	9								
			6-OHDA LD + L-DOPA	299.00	30.91	18	4								
		M4-IPSC amplitude (pA) DMS vs eStim intensity (µA)	eStim intensity x Treatment					Two-Way RM ANOVA with Geisser Greenhouse's correction	F(10, 305)=1.960	p=0.0373	*	Holm-Šídák			
			eStim intensity						F(1.988, 121.3)=190.1	p<0.0001	****				
			Treatment						F(2, 61)=2.142	p=0.1261	n.s.				
			Subject						F(61, 305)=23.61	p<0.0001	****				
		30 µA	Saline	476.41	44.62	17	9						Saline vs. 6OHDA LD	p=0.3762	n.s.
			6-OHDA LD	421.63	41.89	29	9						Saline vs. 6OHDA LD + L-DOPA	p=0.0515	n.s.
			6-OHDA LD + L-DOPA	338.63	31.62	18	4						6OHDA LD vs. 6OHDA LD + L-DOPA	p=0.227	n.s.
		25 µA	Saline	443.57	43.02	17	9						Saline vs. 6OHDA LD	p=0.4231	n.s.
			6-OHDA LD	395.22	41.45	29	9						Saline vs. 6OHDA LD + L-DOPA	p=0.0315	*
			6-OHDA LD + L-DOPA	298.98	30.91	18	4						6OHDA LD vs. 6OHDA LD + L-DOPA	p=0.1337	n.s.
		20 µA	Saline	391.54	41.37	17	9						Saline vs. 6OHDA LD	p=0.4047	n.s.
			6-OHDA LD	344.06	38.26	29	9						Saline vs. 6OHDA LD + L-DOPA	p=0.0292	*
			6-OHDA LD + L-DOPA	253.67	27.70	18	4						6OHDA LD vs. 6OHDA LD + L-DOPA	p=0.1203	n.s.
		15 µA	Saline	316.63	41.34	17	9						Saline vs. 6OHDA LD	p=0.6167	n.s.
			6-OHDA LD	288.69	36.83	29	9						Saline vs. 6OHDA LD + L-DOPA	p=0.1056	n.s.
			6-OHDA LD + L-DOPA	204.61	30.13	18	4						6OHDA LD vs. 6OHDA LD + L-DOPA	p=0.161	n.s.
		10 µA	Saline	209.97	46.56	17	9						Saline vs. 6OHDA LD	p=0.9619	n.s.
			6-OHDA LD	207.18	34.40	29	9						Saline vs. 6OHDA LD + L-DOPA	p=0.5837	n.s.
			6-OHDA LD + L-DOPA	153.50	31.11	18	4						6OHDA LD vs. 6OHDA LD + L-DOPA	p=0.5837	n.s.
		5 µA	Saline	47.39	14.93	17	9						Saline vs. 6OHDA LD	p=0.5536	n.s.
			6-OHDA LD	60.18	15.33	29	9						Saline vs. 6OHDA LD + L-DOPA	p=0.4046	n.s.
			6-OHDA LD + L-DOPA	26.05	8.74	18	4						6OHDA LD vs. 6OHDA LD + L-DOPA	p=0.1692	n.s.
	D	Max Oxo-current (pA) DLS	Saline	529.90	41.70	12	6	Ordinary One-Way ANOVA	F(2, 31)=13.53	p<0.0001	****	Tukey	Saline vs. 6OHDA LD	p=0.001	**
			6-OHDA LD	311.50	36.92	11	10						Saline vs. 6OHDA LD + L-DOPA	p<0.0001	****
			6-OHDA LD + L-DOPA	265.80	37.12	11	4						6OHDA LD vs. 6OHDA LD + L-DOPA	p=0.694	n.s.
		Max Oxo-current (pA) DMS	Saline	521.80	44.79	14	10	Ordinary One-Way ANOVA	F(2, 29)=4.554	p=0.0191	*	Tukey	Saline vs. 6OHDA LD	p=0.8801	n.s.
			6-OHDA LD	488.20	65.72	8	6						Saline vs. 6OHDA LD + L-DOPA	p=0.0174	*
			6-OHDA LD + L-DOPA	330.70	35.22	10	4						6OHDA LD vs. 6OHDA LD + L-DOPA	p=0.1057	n.s.
